# Total delay in treatment among tuberculous meningitis patients in China: a retrospective cohort study

**DOI:** 10.1186/s12879-017-2447-0

**Published:** 2017-05-12

**Authors:** Yu He, Chao Han, Kai-Feng Chang, Mao-Shui Wang, Tian-Ren Huang

**Affiliations:** 1 Department of Clinical Laboratory, First Affiliated Hospital of Guangxi Medical University, Nanning, China; 2 Department of Geriatrics, Shandong Mental Health Center, Jinan, China; 3 Department of Infection Prevention, Pingyi Center for Disease Control and Prevention, Pingyi, China; 4 Department of Lab Medicine, Shandong Provincial Chest Hospital, Jinan, China; 5 Department of Epidemiology, Affiliated Tumor Hospital of Guangxi Medical University, 71#, Hedi Road, Nanning, 530021 China

**Keywords:** Tuberculous meningitis, Factor, Treatment delay

## Abstract

**Background:**

Currently, there is limited data on the risk factors associated with treatment delay in tuberculous meningitis (TBM). This study aimed to assess the duration of delay in the treatment TBM and to investigate its determinants.

**Methods:**

During the period from September 2009 to February 2016, a retrospective cohort study of consecutive TBM patients admitted to our hospital was conducted to determine the risk factors associated with treatment delay in TBM. Treatment delay duration was defined as the time interval from onset of symptoms (by patient recall) to initiation of treatment and was stratified into two categories: ≤ 20 days, >20 days (median delay day is 20 days). Data collected included demography, comorbidity, cerebrospinal fluid (CSF) examinations and others. Univariable and multivariable logistic regression analysis was used to evaluate the determinants of treatment delay.

**Results:**

A total of 161 TBM patients were included in our study, all were confirmed by CSF mycobacterial culture. The median treatment delay for all patients included in the study was 20 days [interquartile range, 14–60 days]. Multivariate analysis revealed that age (≤21 years old, OR = 0.202, 95% CI: 0.079, 0.521), fever (OR = 0.414, 95% CI: 0.180, 0.952), and headache (OR = 0.204, 95% CI: 0.095, 0.442) had significantly lower risk for treatment delay, while multiple healthcare contact (>3 times) (OR = 3.938, 95% CI: 1.326, 11.691) as well as CSF chloride (>111 mmol/L) (OR = 2.479, 95% CI: 1.143, 5.377) had significantly higher risk of the delay.

**Conclusions:**

Our findings indicate that multiple healthcare contact and high CSF chloride predict the risk of long delay, while young age, fever and headache are associated with short delay. Maintained focus on awareness of TBM in the population and in healthcare systems, and continuous implementation of diagnostic methods for TBM to detect the disease early, may reduce the mortality and morbidity.

## Background

Tuberculosis (TB) remains a severe global public health threat. In 2014, 9.6 million people are estimated to have fallen ill with TB, and 1.5 million people died from TB [1]. TB usually affects the lungs, but it also can affect any other organ of the body, e.g. lymph nodes, pleura, or meninges. Tuberculous meningitis (TBM) occurs in approximately 1% of all patients with active TB, but is disproportionately important because it kills or severely disables about half of the people affected [2].

TBM is the most severe form of TB and a medical emergency. A number of factors have been reported to be associated with poor outcome. Some of these factors are diagnosis delay; severity of TBM at the time of admission; the presence of headache, fever and hydrocephalus; high cerebrospinal fluid (CSF) protein and lactate concentration [3, 4]. Rapid diagnosis and early treatment before the occurrence of progression of stage are crucial for the outcome of TBM. A study conducted in Taiwan, showed that 47.6% of patients experienced delay in initiating appropriate treatment [3]. Until now, several studies have proved that treatment delay is strongly associated with poor outcomes in adults or children [2, 5–9].

Factors affecting treatment delay are of key importance in TB management as delay may increase mortality [10]. Understanding the factors related to delay in treatment of disease is essential to reduce morbidity and mortality of TBM, and decreases the risk of mortality. However, there is limited data on the risk factors associated with treatment delay. This study aimed to assess the duration of delay in the treatment of TBM and to investigate its determinants. The findings of our study may be useful for management of TBM in the field of improving diagnosis, increasing physician awareness, control planning and policies in China.

## Methods

### Study design and population

This study was approved by the Human Research Ethics Committees of Shandong Provincial Chest Hospital (SPCH) and First Affiliated Hospital of Guangxi Medical University. This retrospective study was carried out in Jinan city, China during the period from September 2009 to February 2016.

We conducted a retrospective cohort study of consecutive TBM patients admitted to SPCH to determine the risk factors associated with treatment delay. TBM was defined as positive CSF mycobacterial culture. Delay was measured in days. Treatment delay duration was defined as the time interval from onset of symptoms (by patient recall) to initiation of treatment and was stratified into two categories: ≤ 20 days (short delay), >20 days (long delay).

### Data collection

Data were collected by a questionnaire filled out by accessing medical records. Data collected included (1) demography (gender, age, symptoms (fever, headache, cough, vomiting, coma), previous anti-tuberculosis therapy, smoking habit, contact history of TB, hydrocephalus, and time during hospitaliziation); (2) comorbidity (pulmonary TB, extrapulmonary TB, diabetes mellitus, rheumatic diseases, miliary TB, recurrent TB); (3) CSF examinations (white blood cell (WBC), mononuclear cells, polynuclear cells, glucose, lactate, tryptophan, aspartate transaminase, lactate dehydrogenase, total protein, α-hydrooxybutyrate dehyrogenase, chloride, adenosine deaminase); (4) others (serum glucose).

### Data analysis

Patient characteristics were summarised using means and standard deviations for continuous variables and counts/percentages for categorical variables. Univariate analysis was carried out using the χ2 test or Fisher exact test for categorical data and the Mann-Whitney *U* test for continuous variables. Beyond descriptive statistics, associations between the dependent variables (treatment delay) and the independent variables were analyzed by calculating the Odds Ratios (OR) and 95% confidence interval (CI). Multivariate logistic regression analysis was used to evaluate risk factors for the delay, using selection of factors associated with delay in univariate analysis (*P* < 0.1) or those known to have clinical significance. The Hosmer-Lemeshow goodness-of-fit test was performed to assess the overall fit of the model. A two-sided *P*-value <0.05 was considered significant for all analyses. Data analysis was carried out using SPSS 18.0 (IBM Corp., Armonk, United States).

## Results

### Baseline patient characteristics

Table 1 shows the characteristics of the participants of this study. A total of 161 TBM patients were included in our study, all were confirmed by CSF mycobacterial culture. Males comprised 54.7% of the participants. Smokers constituted 16.8% of participants. Nineteen patients had anti-TB therapy before admission to our hospital, and 18 patients had contact of TB history. Among them, 109 (67.7%) participants had pulmonary tuberculosis, 48 (29.8%) had extrapulmonary tuberculosis (excluding TBM), 8 had rheumatic diseases, 11 had diabetes mellitus, 51 had millary TB, 12 had recurrent TB. Fever was the most frequently reported symptom (72.0%) followed by headache (55.9%), vomiting (19.3%), cough (18.6%) and hydrocephalus (11.2%). Twenty five patients (15.5%) were diagnosed after one health care contact, 63 (39.1%) after two visits, 48 (29.8%) after three visits, 17 (10.6%) after four visits, and 8 (5.0%) after five or more visits. Results of CSF examinations are also summarized in Table 1.

**Table 1:** Univariate analysis of risk factors associated with treatment delay in TBM patients

	Short delay	Long delay	Total	*P* value
N	84	77	161	
Age (years)	28.3 ± 16.9	34.1 ± 17.2	31.1 ± 17.2	0.037
Sex (male)	48	40	88	0.509
Time during hospitalization (days)	56.6 ± 44.6	64.6 ± 47.1	60.4 ± 45.9	0.273
Fees ($)	78,721 ± 71,922	98,186 ± 108,770	88,030 ± 91,645	0.192
Previous anti-TB therapy	5	14	19	0.022
Smoking habit (pack-years)	2.82 ± 9.10	3.36 ± 11.9	3.08 ± 10.50	0.746
Contact history of TB	10	8	18	0.761
No. of Healthcare visits	2.3 ± 0.9	2.8 ± 1.3	2.5 ± 1.1	0.010
Comorbidity				
Pulmonary TB	54	55	109	0.334
Extrapulmonary TB	23	25	48	0.481
Rheumatic diseases	3	5	8	0.401
Diabetes mellinus	3	8	11	0.434
Milliary TB	22	29	51	0.120
Recurrent TB	3	9	12	0.064
Symptoms				
Fever	68	48	116	0.010
Headache	63	27	90	0.000
Vomitting	23	8	31	0.008
Cough	10	20	30	0.025
Hydrocephalus	13	5	18	0.147
Abnormalty in brain CT	37	27	64	0.941
CSF examination				
WBC (10^9^/L)	207 ± 210	187 ± 183	198 ± 198	0.524
Mononuclear cells (%)	64 ± 28	54 ± 28	59 ± 29	0.041
Polynuclear cells (%)	36 ± 29	46 ± 28	41 ± 28	0.037
Glucose (mmol/L)	1.96 ± 1.21	2.26 ± 1.81	2.11 ± 1.53	0.223
Tryptophan (+)	59	49	108	0.708
Lactate (mmol/L)	6.75 ± 2.37	6.40 ± 2.43	6.58 ± 2.39	0.410
AST (U/L)	18.7 ± 16.5	17.8 ± 10.5	18.3 ± 13.9	0.671
LDH (U/L)	136 ± 209	120 ± 138	129 ± 178	0.569
CSF-PR (mg/L)	1323 ± 649	1456 ± 758	1387 ± 704	0.230
α-HBDH (U/L)	131 ± 402	70 ± 73	102 ± 296	0.257
Chloride (mmol/L)	108 ± 8	111 ± 8	109 ± 8	0.038
ADA (U/L)	8.1 ± 8.1	8.5 ± 6.4	8.3 ± 7.3	0.738
CSF/Serum glucose	0.32 ± 0.18	0.32 ± 0.18	0.32 ± 0.18	0.932
Serum glucose (mmol/L)	6.30 ± 1.54	7.07 ± 3.27	6.66 ± 2.53	0.084

### Treatment delay

The median treatment delay for all patients included in the study was 20 days (interquartile range, 14–60 days). For 77 (47.8%) patients, the treatment delay was >20 days. Table 1 shows the univariate analysis on risk factors, comparing patients with total delay of >20 days with patients with total delay of ≤20 days. The delay was associated with age, previous anti-TB therapy, multiple healthcare contact, symptoms (fever, headache, vomiting and cough), and CSF examinations (mononuclear and polynuclear cell concentration, and chloride) (all *P* < 0.05) (Table 1).

Further multivariate analysis revealed that age (≤21 years old, OR = 0.202, 95% CI: 0.079, 0.521), fever (OR = 0.414, 95% CI: 0.180, 0.952), and headache (OR = 0.204, 95% CI: 0.095, 0.442) had significantly lower risk for treatment delay, while multiple healthcare contact (>3 times) (OR = 3.938, 95% CI: 1.326, 11.691) as well as CSF chloride (>111 mmol/L) (OR = 2.479, 95% CI: 1.143, 5.377) had significantly higher risk of the delay (Table 2).

**Table 2:** Multivariate analysis of risk factors associated with treatment delay in TBM patients

	*P* value	OR	95% CI
Age (≤21 years old)	0.001	0.202	0.079, 0.521
Fever	0.038	0.414	0.180, 0.952
Headache	0.000	0.204	0.095, 0.442
No. of Healthcare visits (>3 times)	0.014	3.938	1.326, 11.691
Chloride (>111 mmol/L)	0.022	2.479	1.143, 5.377

## Discussion

Although TBM is a medical emergency, our study shows that a significant delay in treatment among TBM patients. Here, we found a median treatment delay time of 20 days in TBM patients. Early detection of cases and treating TBM patients are one of the strategies to reduce the disease’s morbidity and mortality throughout the world. The treatment delay in TBM can be divided into the length of patient delay and the diagnosis delay [11]. Identifying the sources of delay is a critical issue for TBM therapy. In our study, the main factors associated with treatment delay in multivariate analysis were age (≤21 years old, OR = 0.202), fever (OR = 0.414), headache (OR = 0.204), multiple healthcare contact (OR = 3.938), and CSF chloride (>111 mmol/L, OR = 2.479). These may be likely explained by three main factors: perceived severity of illness and symptom recognition, lack of specific and rapid diagnostic methods in TBM and quality of health service provision.

Firstly, in the study, we found a clear association between treatment delay and clinical symptoms at inclusion, which has not been described before. Our results showed that symptoms, including fever and headache, were protective factors for shortening treatment delay. The two, headache and fever, can be a sign of infection that is localized to central nervous system, which consists of brain and spinal cord, such as meningitis, encephalitis, and a brain abscess. The combination may raise awareness of TBM among them. Younger age was also a protective factor for shortening the delay. This may be explained by the two reasons: 1) student is free of economic pressures, he can spend more time seeking health care; 2) in China, the One-child Policy forced parents to cherish their only child, and it saves much time in seeking health care for their children.

Secondly, diagnosing TBM remains a challenge. The early diagnosis of TBM is fundamental to avoiding treatment delay. However, it is always difficult to confirm the clinical suspicion of TBM. TBM presents in a non-specific manner and it is rare to confirm the diagnosis microbiologically even in high resource settings. Currently, there is no single test with optimal sensitivity in patients with suspected TBM. Routine assays, i.e., acid-fast bacillus staining and mycobacterial culture, are insensitive and slow. It was reported that only 10 to 20% of TBM patients were detected by CSF AFB staining [12], while other tests like Xpert MTB/RIF have several limitations including high cost, requirement for a stable electricity supply and short shelf life of consumables [13]. CSF adenosine deaminase has been evaluated and may be used as a supportive test in diagnosis, but is not recommended as a routine diagnostic test for TBM [2, 14, 15].

In our study, CSF chloride (>111 mmol/L) had significantly higher risk of the delay. This may be associated with the two reasons: 1) In practice, once the diagnosis of bacterial meningitis has been excluded, uncommonly low CSF chloride values (<110 mmol/L) may be associated with TBM but not with viral meningitis [16]. Therefore, TBM may be rapid and timely diagnosed relying on the rule by practitioners; 2) decreased CSF chloride was associated with increased intracranial pressure. This may be helpful to shorten the time between symptom onset and first visit to any health care provider.

Thirdly, the mean number of visits to health care facilities before the diagnosis was 2.5. This finding could be related to diagnostic challenge in TBM and lack of awareness of TB among health care workers. Under these circumstances, it is easy to neglect many important issues in TBM diagnosis and management, resulting from a lack of experience and expertise which had previously been found to be associated with diagnosis delays [17].

Moreover, in China, poverty has been reported as central factor causing delay in health care seeking [18–20]. Among the TB patients identified in the 4th National TB Survey, 37% ascribed their delays in clinical consultation to financial problems [21]. A majority of health providers believe there would be improved TB treatment compliance and overall control of TB infections if there was a decreased financial burden for patients and families [11]. Besides extent of the free TB diagnosis and treatment, educational attainment and knowledge of TB possibly enhances detection of TBM and shorten diagnosis delays [11].

This study has potential limitations. First, there is no agreed definition of what constitutes an “acceptable” delay. It probably depends on the local health services and the local epidemiological situation, with a shorter delay to be expected when incidence is high [22]. In our study, we used a cut-off interval of 20 days for subsequent analysis which was similar to that found by other authors in TB [7, 23–25]. Second, considering that it was difficult to access to the results of previous CSF examinations and remarkable difference existed in the principles of CSF assays between hospitals, these results of CSF examinations performed at our hospital were used, which may have overestimated the clinical severity. Third, TB is a chronic disease with an insidious start, making it difficult for patients to remember exactly when the symptoms started. Thus, recall bias may occur. Fourth, the results of our study need to be carefully interpreted because this data was based on a single hospital experience, which was not enough to represent the characteristics of TBM in the entire Chinese population. In addition, although several risk factors were discovered, additional studies are required to validate our findings.

## Conclusions

Our findings represent a population-based analysis over a long time period and are based on a large dataset. The results clearly show that total delay in treatment of TBM is still high in the study area. Our findings indicate that multiple healthcare contact and high CSF chloride predict the risk of long delay, while younger age, fever and headache are associated with short delay. Maintained focus on awareness of TBM in the population and in healthcare systems and continuous implementation of diagnostic methods for TBM to detect the disease early, may reduce the mortality and morbidity.

## Abbreviations

CI: Confidence interval
CSF: Cerebrospinal fluid
OR: Odds ratios
TB: Tuberculosis
TBM: Tuberculous meningitis
WBC: White blood cell
